# Association between neutrophil to lymphocyte ratio and all-cause mortality in critical patients with coronary artery disease - a study based on the MIMIC-IV database

**DOI:** 10.3389/fcvm.2025.1502964

**Published:** 2025-03-21

**Authors:** Yao Li, Dongbo Chen, Yifei Fan, Qing Zhu, Han Deng, Xin Chai

**Affiliations:** ^1^ Department of Critical Care Medicine, Xijing Hospital, Fourth Military Medical University, Xi’an, Shaanxi, China; ^2^ Institute of Medical Research, Northwestern Polytechnical University, Xi'an, Shaanxi, China; ^3^ Department of General Surgery, Xi'an Eighth Hospital, Xi'an, Shaanxi, China

**Keywords:** coronary artery disease, neutrophil-to-lymphocyte ratio (NLR), MIMIC-IV, all-cause mortality, ICU

## Abstract

**Background:**

Neutrophil-to-lymphocyte ratio (NLR) has been presented as a possible indicator associated with the outcomes of growing patients and an available predictor of inflammation. Nevertheless, just a handful of researches shed light on the association between NLR and the consequences of critical patients with coronary artery disease (CAD). The study aimed to investigate the correlation between NLR and all-cause mortality of short-term and long-term in patients with CAD.

**Methods:**

We obtained objective data from the Medical Information Mart for Intensive Care (MIMIC)-IV version 2.2, a comprehensive and large-scale single-center database. NLR was calculated separately. Patients were categorized by quartiles of NLR: Q1 group (NLR < 3.56), Q2 (NLR 3.56–5.54), Q3 group (NLR 5.54–9.05), Q4 group (NLR > 9.05). Kaplan–Meier survival curves based on NLR quartiles were created to compare all-cause mortality rates, and the log-rank test evaluated the differences between groups. The hazard ratio (HR) of NLR as a risk factor for outcome events was assessed using the Cox proportional risk model with the Q1 group serving as the reference group and restricted cubic spline (RCS) with the infection points of 5.54.

**Results:**

A total of 3,692 patients were included in this study. The 30-day mortality rate among the patients was 8.85%, while the 365-day mortality rate was 16.98%. High NLR (NLR > 5.54) was significantly associated with 30-day mortality [HR, 3.99,95% confident interval (CI), (3.03–5.24); *P* < 0.001] and 365-day mortality [HR, 5.72, 95% CI (3.83–8.54); *P* < 0.001] in patients with critical CAD in the completely adjusted Cox proportional risk model. RCS analysis revealed a U-shaped relationship between NLR and outcome events.

**Conclusion:**

In patients diagnosed with critical CAD, a significant correlation was observed between NLR and all-cause mortality, particularly among individuals exhibiting elevated NLR levels. These findings suggest that NLR may serve as a valuable prognostic marker for evaluating both short-term and long-term mortality risk in this patient population.

## Introduction

Coronary artery disease (CAD) remains a significant global public health challenge, despite advances in prevention and treatment. Critically ill patients admitted to the intensive care unit (ICU) following cardiac surgery often have CAD, requiring specialized ICU care. Additionally, acute exacerbations of chronic cardiovascular diseases contribute to a notable mortality rate, making cardiovascular issues the second leading cause of death globally within a year (1–5). Evaluating the prognosis of patients with critical CAD is *a priori*ty due to limited current research in this area.

Inflammation plays a crucial role in the development of severe cardiovascular diseases. Inflammatory markers like white blood cell (WBC), C-reactive protein (CRP), and interleukin-6 (IL-6) are strongly linked to cardiovascular disease. Recently, anti-inflammatory drugs have emerged as potential treatments for these patients (6, 7). The ratio of leukocyte subtypes serves as an indicator of inflammatory onset, with NLR emerging as a novel biomarker. Neutrophils signify non-specific inflammatory responses, while lymphocytes reflect stress levels or compromised immune function (8). Severe inflammation adversely affects patients in coronary care units (CCUs) (9), and the NLR has been shown to predict poor outcomes across various diseases. For patients with advanced heart failure, a higher NLR (indicative of a reduced lymphocyte ratio) correlates with poorer long-term outcomes (10). Studies also associate elevated NLR with adverse prognoses in acute coronary syndrome (ACS) and patients undergoing percutaneous coronary treatments (11, 12). However, whether NLR predicts short-term, long-term, or both forms of mortality in CAD patients remains unclear. This study investigates the relationship between NLR and outcomes in CAD patients, aiming to clarify its predictive value.

## Methods and materials

### Data source

**Figure 1:**
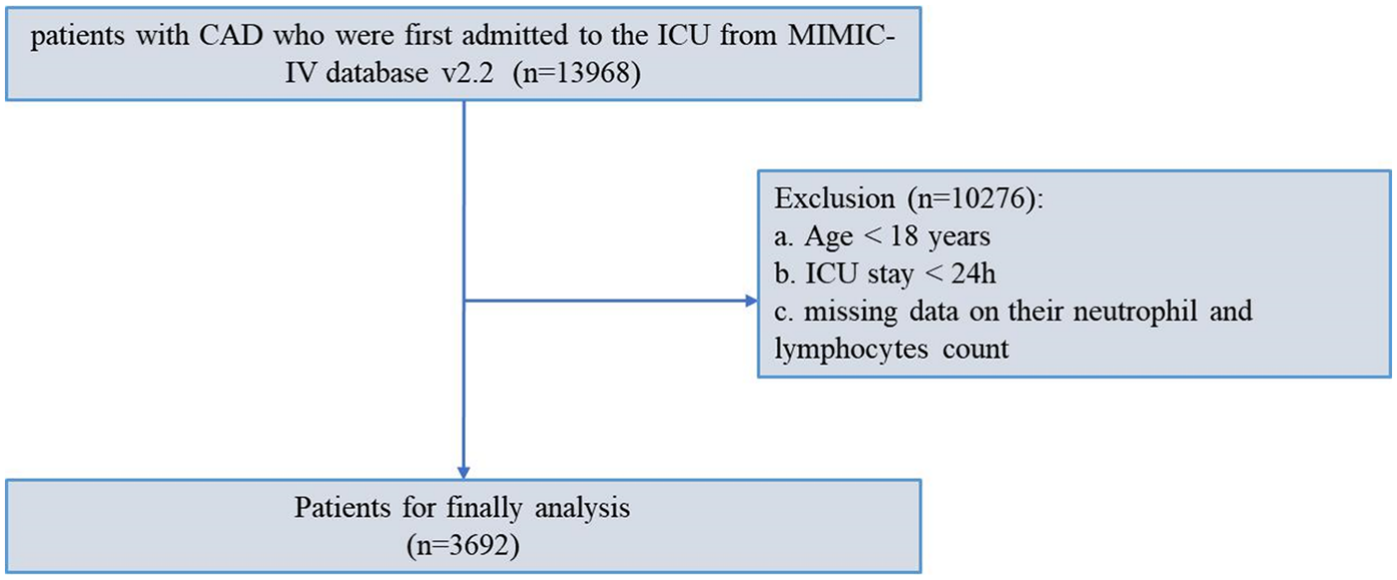
Flowchart of patient inclusion and exclusion from the MIMIC-IV database.

This study involved a retrospective analysis of coronary artery disease patient data from the MIMIC-IV database, managed by MIT's (Massachusetts Institute of Technology) Laboratory of Computational Physiology. The research database received approval from the review committee at the Massachusetts Institute of Technology and Beth Israel Deaconess Medical Center, along with a waiver of informed consent. We have completed the online course and passed the online exams (No. 12892471) to gain access to the database. In this investigation, we enrolled 13,968 patients with critical coronary artery disease who were first admitted to ICU from 2008 to 2019. A total of 10,276 patients were initially excluded due to missing data on their neutrophil and lymphocytes count level. Ultimately, a total of 3,692 patients with critical CAD met the inclusion criteria for the study (Figure 1).

### Data extractions

The extraction of information was facilitated by the utilization of PostgresSQL (version 13.7.2) and Navicate Premium (version 16) software, employing the execution of Structured Query Language (SQL). Potential confounding variables were extracted as follows: (1). baseline demographic information: age, gender, body mass index (BMI), (2). comorbidities: hypertension, diabetes, atrial fibrillation (AF), acute myocardial infarction (AMI), acute heart failure (AHF), (3). interventions: coronary artery bypass grafting (CABG), percutaneous transluminal coronary angioplasty (PTCA), invasive ventilation, (4). laboratory parameters: white blood cells (WBC), red blood cells(RBC), hemoglobin(HGB), platelets(PLT), Hematocrit (Hct), Sodium, Potassium, calcium, glucose and Red cell distribution (RDW), (5). Scoring system: Acute Physiology Score III (APSIII), and Sepsis-Organ Failure Assessment Score (SOFA). Simpliﬁed Acute Physiology Score (SAPS II), Oxford Acute Severity of Illness Score (OASIS) and Charlson comorbidity index. All variables were complete except for serologic indicators. The missing serological values are mainly concentrated in the following variables(missing data and percent): Platelet count: 2(0.05%), hemoglobin:1(0.03%), potassium: 9(0.24%), fasting plasma glucose:48(1.3%). We used the random forest method to impute missing serologic values, as they were less than 5%.

The baseline NLR was calculated by dividing the absolute neutrophil count by the absolute lymphocyte count using the initial laboratory parameters following admission to the ICU. Subsequent assessments of NLR were conducted with the maximum value within the first week of ICU admission, with measurements taken at irregular intervals. The main outcome measure in this study was all-cause mortality occurring within 365 days following patient admission, with the secondary outcome defined as all-cause mortality within 30 days post-admission.

### Statistical analysis

NLR quartiles were used to categorize the study population into four groups: Q1 (*n* = 923, NLR ≤ 3.56), Q2 (*n* = 923, 3.56 < NLR ≤ 5.54), Q3 (*n* = 923, 5.54 < NLR ≤ 9.05), and Q4 (*n* = 923, NLR > 9.05), with the highest two quartiles classified as high group and the lower two quartiles classified as low group. Categorical variables were represented as percentages and compared using the chi-square test, while continuous numerical variables were expressed as medians (quartiles) following a normality test and compared using the nonparametric rank-sum test. The HR of NLR as a risk factor for outcome events was evaluated using a Cox proportional risk model with the Q1 group as the reference. Age, gender, BMI, PTCA, CABG, AMI, AHF, AF, hypertension, diabetes and invasive ventilation therapy were considered as confounders in the multivariate Cox regression model. Kaplan–Meier survival analysis based on NLR quartiles and the log-rank test were used to compare groups. Restricted cubic spline (RCS) curves were utilized to investigate the relationship between NLR and outcome events. Subgroup analysis were conducted to validate the reliability of the findings. Receiver operating characteristic curves were used to assess the prognostic value of NLR for 30-day and 365-day mortality. Statistical analysis was carried out using R studio (version R4.2.3) and EmpowerStats (version 4.1), with a two-sided *P* value < 0.05 considered statistically significant.

## Results

### Baseline information of patients

This study encompassed a cohort of 3,692 patients diagnosed with critical coronary artery disease, consisting of 2,748 male patients (74.4%) and 944 female patients (25.6%,), among whom 627 patients encountered a fatal event within the 365-day observational period. The baseline information based on the NLR quartiles is shown in Table 1. In comparison to the low-NLR group, the high-NLR group exhibited a lower proportion of young male patients, and elevated percentages of CABG procedures, PLT count, and creatinine levels. Conversely, the low-NLR group demonstrated higher percentages of patients with AMI, AHF, AF and elevated hemoglobin levels. Furthermore, the high NLR group displayed higher disease severity scores (SOFA score, APSIII score, SAPSII score, OASIS and Charlson) and WBC counts compared to the low NLR group. Baseline characteristics difference between survivors and non-survivors during the hospital stay are shown in Supplementary Table S1.

**Table 1:** Baseline information of patients.

Variables	Q1 (*N* = 923)	Q2 (*N* = 923)	Q3 (*N* = 923)	Q4 (*N* = 923)	Total (*N* = 3,692)	*P-*value
Demographics						
Age (%)	69.7 ± 10.8	69.2 ± 10.5	70.1 ± 10.9	73.5 ± 11.0	70.6 ± 10.9	<0.001
>65	631 (68.4%)	630 (68.3%)	662 (71.7%)	732 (79.3%)	2,655 (71.9%)	
Gender						<0.001
Male	659 (71.4%)	698 (75.6%)	730 (79.1%)	661 (71.6%)	2,748 (74.4%)	
Female	264 (28.6%)	225 (24.4%)	193 (20.9%)	262 (28.4%)	944 (25.6%)	
Ethnicity						<0.001
White	594 (64.4%)	624 (67.6%)	680 (73.7%)	658 (71.3%)	2,556 (69.2%)	
Black	199 (21.7%)	212 (23%)	189 (20.5%)	202 (21.9%)	802 (21.7%)	
Asian	30 (3.2%)	17 (1.8%)	9 (1%)	20 (2.1%)	76 (2.1%)	
Hispanic	65 (7%)	48 (5.2%)	30 (3.2%)	30 (3.3%)	173 (4.7%)	
Others	35 (3.7%)	22 (2.4%)	15 (1.6%)	13 (1.4%)	85 (2.3%)	
Laboratory tests						
Neutrophil count	6.7 ± 3.5	9.5 ± 3.6	11.1 ± 4.5	13.9 ± 7.0	10.3 ± 5.5	<0.001
Lymphocytes	3.2 ± 9.3	2.1 ± 0.8	1.6 ± 0.6	0.9 ± 0.5	2.0 ± 4.7	<0.001
NLR	2.5 ± 0.8	4.5 ± 0.6	7.0 ± 1.0	20.2 ± 17.3	8.6 ± 11.1	<0.001
WBC (×10^−9^/L)	12.2 ± 12.4	13.2 ± 4.3	14.4 ± 6.6	15.7 ± 7.6	13.9 ± 8.4	<0.001
RBC (×10^−9^/L)	3.4 ± 0.6	3.5 ± 0.6	3.4 ± 0.6	3.5 ± 0.7	3.5 ± 0.6	0.017
PLT	154.1 ± 54.7	163.0 ± 57.6	168.0 ± 63.9	192.5 ± 98.2	169.4 ± 72.1	<0.001
HGB	10.2 ± 1.7	10.4 ± 1.5	10.3 ± 1.7	10.4 ± 2.1	10.3 ± 1.8	0.031
RDW (%)	14.0 ± 1.9	13.9 ± 1.8	14.1 ± 2.0	15.1 ± 2.4	14.3 ± 2.1	<0.001
Hct (%)	31.1 ± 4.8	31.7 ± 4.4	31.5 ± 5.0	32.0 ± 6.1	31.6 ± 5.1	0.004
FPG	127.4 ± 38.2	128.6 ± 37.0	134.6 ± 47.4	155.7 ± 70.3	136.6 ± 51.3	<0.001
Scr (mg/dl)	1.2 ± 1.1	1.1 ± 0.8	1.3 ± 1.0	1.7 ± 1.5	1.3 ± 1.2	<0.001
Bun (mg/dl)	19.7 ± 13.7	18.9 ± 11.0	23.1 ± 18.3	33.0 ± 23.9	23.7 ± 18.3	<0.001
Scoring system						
SOFA	5.0 ± 2.7	5.0 ± 2.7	5.3 ± 3.0	6.4 ± 3.8	5.4 ± 3.1	<0.001
APSIII	37.2 ± 16.9	36.9 ± 17.1	40.9 ± 19.6	49.3 ± 21.4	41.1 ± 19.5	<0.001
SAPSII	36.4 ± 10.9	36.7 ± 11.1	38.2 ± 12.4	43.3 ± 13.8	38.7 ± 12.4	<0.001
OASIS	30.6 ± 7.5	30.6 ± 6.9	31.1 ± 7.9	33.9 ± 8.7	31.6 ± 7.9	<0.001
Charlson	5.0 ± 2.5	4.7 ± 2.5	5.2 ± 2.7	6.6 ± 2.8	5.4 ± 2.7	<0.001
Comorbidities						
Hypertension (%)						<0.001
No	407 (44.1%)	424 (45.9%)	471 (51%)	597 (64.7%)	1,899 (51.4%)	
Yes	516 (55.9%)	499 (54.1%)	452 (49%)	326 (35.3%)	1,793 (48.6%)	
Diabetes (%)						0.003
No	498 (54%)	556 (60.2%)	563 (61%)	567 (61.4%)	2,184 (59.2%)	
Yes	425 (46%)	367 (39.8%)	360 (39%)	356 (38.6%)	1,508 (40.8%)	
AHF						<0.001
No	696 (75.4%)	686 (74.3%)	628 (68%)	458 (49.6%)	2,468 (66.8%)	
Yes	227 (24.6%)	237 (25.7%)	295 (32%)	465 (50.4%)	1,224 (33.2%)	
AMI						<0.001
No	705 (76.4%)	690 (74.8%)	664 (71.9%)	588 (63.7%)	2,647 (71.7%)	
Yes	218 (23.6%)	233 (25.2%)	259 (28.1%)	335 (36.3%)	1,045 (28.3%)	
AF						0.001
No	601 (65.1%)	549 (59.5%)	542 (58.7%)	520 (56.3%)	2,212 (59.9%)	
Yes	322 (34.9%)	374 (40.5%)	381 (41.3%)	403 (43.7%)	1,480 (40.1%)	
Interventions						
Invasive ventilation						<0.001
No	920 (99.7%)	918 (99.5%)	913 (98.9%)	903 (97.8%)	3,654 (99%)	
Yes	3 (0.3%)	5 (0.5%)	10 (1.1%)	20 (2.2%)	38 (1%)	
CABG (%)						<0.001
No	600 (65%)	584 (63.3%)	666 (72.2%)	814 (88.2%)	2,664 (72.2%)	
Yes	323 (35%)	339 (36.7%)	257 (27.8%)	109 (11.8%)	1,028 (27.8%)	
PTCA (%)						0.207
No	921 (99.8%)	919 (99.6%)	922 (99.9%)	917 (99.3%)	3,679 (99.6%)	
Yes	2 (0.2%)	4 (0.4%)	1 (0.1%)	6 (0.7%)	13 (0.4%)	
Outcomes						
Hospital day	9.0 ± 9.4	8.6 ± 6.5	9.3 ± 7.5	11.0 ± 9.5	9.5 ± 8.4	<0.001
ICU day	2.5 ± 3.1	2.7 ± 3.8	3.0 ± 4.1	4.1 ± 5.5	3.1 ± 4.3	<0.001

Data are expressed in *n* (%) and median (inter-quartile range).

CABG, coronary artery bypass grafting; PTCA, percutaneous transluminal coronary angioplasty; AMI, acute myocardial infarction; AHF, acute heart failure; AF, atrial fibrillation; SOFA, sepsis-organ failure assessment score; APSIII, acute physiology score III; SAPSII, simplified acute physiology score; OASIS, Oxford acute severity of illness score; WBC, white blood cells; RBC, red blood cells; PLT, platelets; HGB, hemoglobin; FPG, fasting plasma glucose; Hct, hematocrit; Scr, creatinine; BUN, ureanitrogen.

### Survival analysis

**Figure 2:**
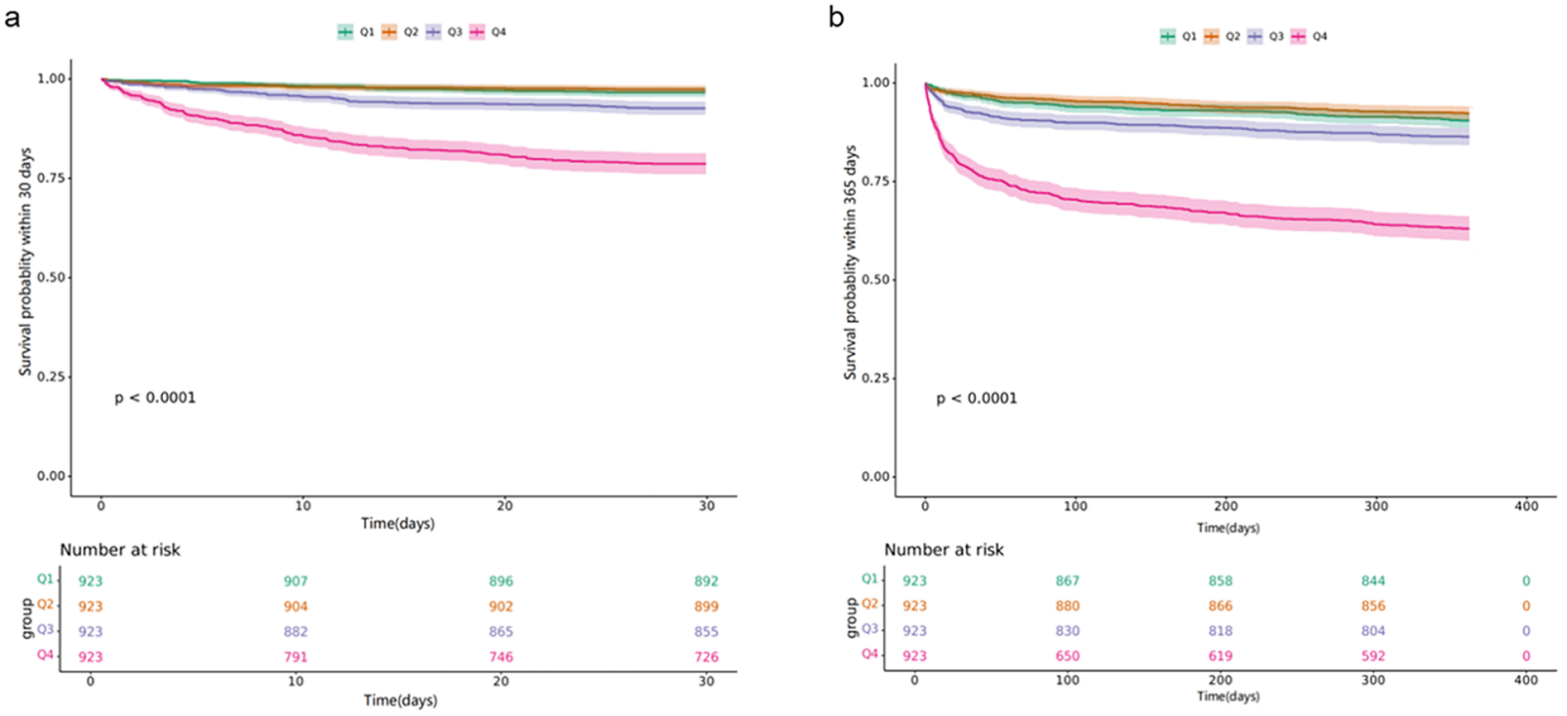
Kaplan–meier all-cause mortality survival analysis curve: **(a)** showing comparison of mortality within 30 days between groups, **(b)** showing comparison of mortality within 365 days between groups.

We conducted a comparison of the incidence of the primary outcome among groups using KM survival analysis curves based on the NLR quartiles, as depicted in Figure 2. The rate of mortality within 30 days was significantly higher in the Q4 group compared to the other groups (log-rank *P* < 0.001) (Figure 2a). Further KM survival analysis were performed between every two groups (Supplementary Figure S1). Additionally, the one-year mortality rate was significantly higher in Group Q4 compared to other groups, with a significant difference between the groups (log-rank *P* < 0.001) (Figure 2b). These findings suggest that high NLR levels have a detrimental impact on the long-term survival of patients with CAD.

### Correlation between NLR and outcome events

In the analysis of patients' baseline characteristics, it was observed that the Q1 group (NLR < 3.56) exhibited the lowest mortality rate in comparison to the other groups. Subsequently, a correlation between NLR and the primary outcome was examined through the development of Cox proportional risk models, with the Q1 group serving as the reference category. The findings revealed that the Q3 and Q4 group (NLR > 9.05) demonstrated a significant association with both the primary outcome event [Q4 vs. Q1: HR, 8.11 [5.49, 11.99], *P* < 0.001; Q3 vs. Q1: HR, 2.32 [1.51, 3.59]] and the secondary outcome [Q4 vs. Q1: HR, 5.56 [4.30, 7.19], *P* < 0.001; Q3 vs. Q1: HR, 1.51 [1.13, 2.02], *P* = 0.005] in the Cox proportional risk model unadjusted for confounders. We observed a significant association between NLR and increased 365-day mortality [Q4 vs. Q1: HR, 5.72 (3.83, 8.54) *P* < 0.001] and 30-day mortality [Q4 vs. Q1: HR, 3.99(3.03, 5.24) *P* < 0.001] in fully adjusted models. Furthermore, we observed a correlation between Q3 group with 5.54 < NLR ≤ 9.05 and 30-day mortality [Q3 vs. Q1: HR, 1.40 (1.04, 1.90) *P* = 0.028], as well with 365-day mortality [Q3 vs. Q1: HR, 2013 (1.37, 3.32) *P* < 0.001], in the fully adjusted model. Moreover, the risk of cardiovascular events was not significant between quartiles 1 and 2 groups both in the unadjusted and fully adjusted model (Table 2).

**Table 2:** Correlation between NLR and outcome events.

Variables	Model 1		Model 2		Model 3	
	HR (95% CI)	*P*	HR (95% CI)	*P*	HR (95% CI)	*P*
All-cause mortality within 30 days						
Q1	1.00 (Reference)		1.00 (Reference)		1.00 (Reference)	
Q2	0.79 (0.57–1.10)	0.159	0.83 (0.59–1.15)	0.262	0.80 (0.57–1.13)	0.206
Q3	1.51 (1.13–2.02)	**0**.**005**	1.55 (1.15–2.08)	**0**.**004**	1.40 (1.04–1.90)	**0**.**028**
Q4	5.56 (4.30–7.19)	**<**.**001**	4.99 (3.84–6.49)	**<**.**001**	3.99 (3.03–5.24)	**<**.**001**
All-cause mortality within 365 days						
Q1	1.00 (Reference)		1.00 (Reference)		1.00 (Reference)	
Q2	0.77 (0.45–1.32)	0.339	0.80 (0.46–1.37)	0.417	0.77 (0.45–1.33)	0.356
Q3	2.32 (1.51–3.59)	**<**.**001**	2.36 (1.53–3.66)	**<**.**001**	2.13 (1.37–3.32)	**<**.**001**
Q4	8.11 (5.49–11.99)	**<**.**001**	7.20 (4.85–10.68)	**<**.**001**	5.72 (3.83–8.54)	**<**.**001**

HR, hazard ratio; CI, confidence interval.

Model 1: Crude.

Model 2: Adjust: age, gender, BMI.

Model 3: Adjust: age, gender, BMI, CABG, PTCA, AMI, AHF, AF, Hypertension, Diabetes, Invasive ventilation. The bold *p*-values represent <0.05, indicating statistically significant results.

**Figure 3:**
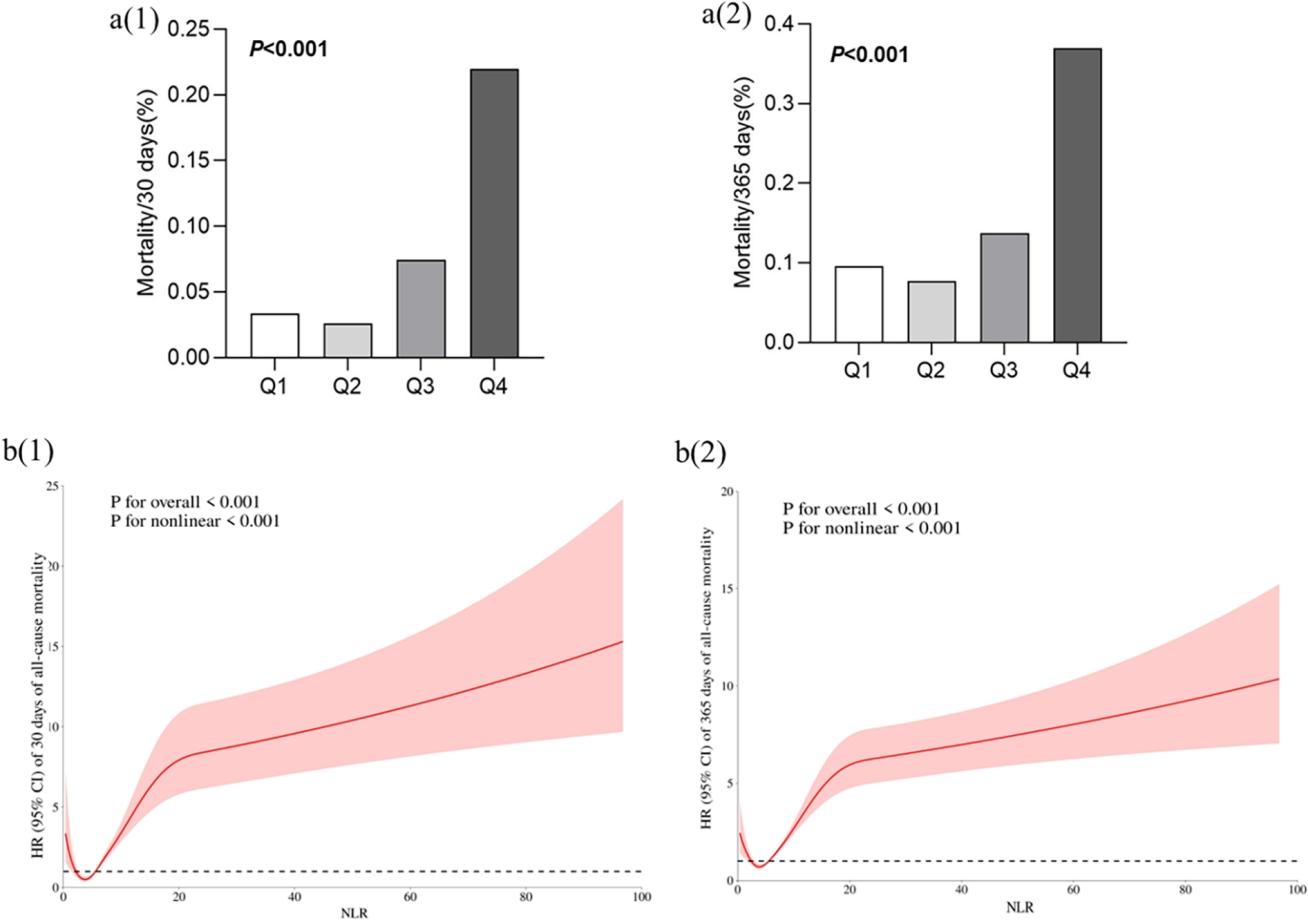
Correlation between NLR and outcome events. **(a)** Comparison of all-cause mortality between groups based on NLR quartiles. **(b)** Restricted cubic spline curve for the NLR index hazard ratio. Heavy central lines represent the estimated fully adjusted hazard ratios for covariates as in Table 2, with shaded ribbons denoting 95% confidence intervals. The horizontal dotted lines represent the hazard ratio of 1.0. **(b)** (1): Restricted Cubic Spline Curve for the mortality rate of patients within 30 days, **(b)** (2): Restricted Cubic Spline Curve for the mortality rate of patients within 365 days.

The data presented in Figure 3a indicates that the Q4 group exhibited the highest mortality rate, with the Q3 group following closely behind. Consequently, it can be inferred that elevated levels of high NLR are associated with increased risk for patients diagnosed with critical coronary heart disease. Following this, we conducted a model of RCS, which revealed a significant “U” shaped relationship between NLR and the risk ratio of mortality in individuals diagnosed with severe coronary artery disease, and we found that NLR infection points of 5.54 for both the primary and secondary outcomes.

### Subgroup analysis

**Figure 4:**
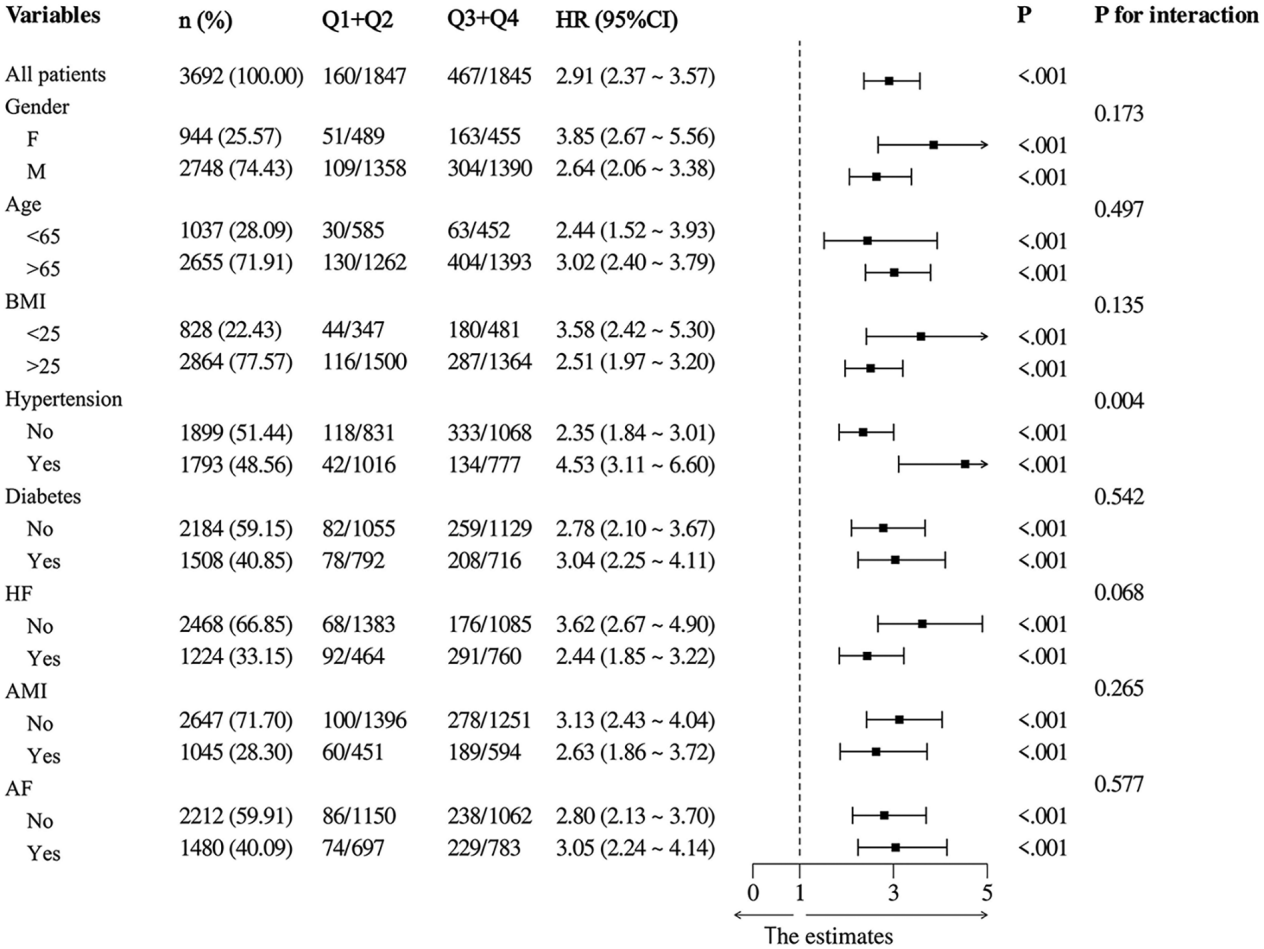
Subgroup analysis with 30-mortality as the outcome event in low and high NLR group (Q1 + Q2 vs. Q3 + Q4).

**Figure 5:**
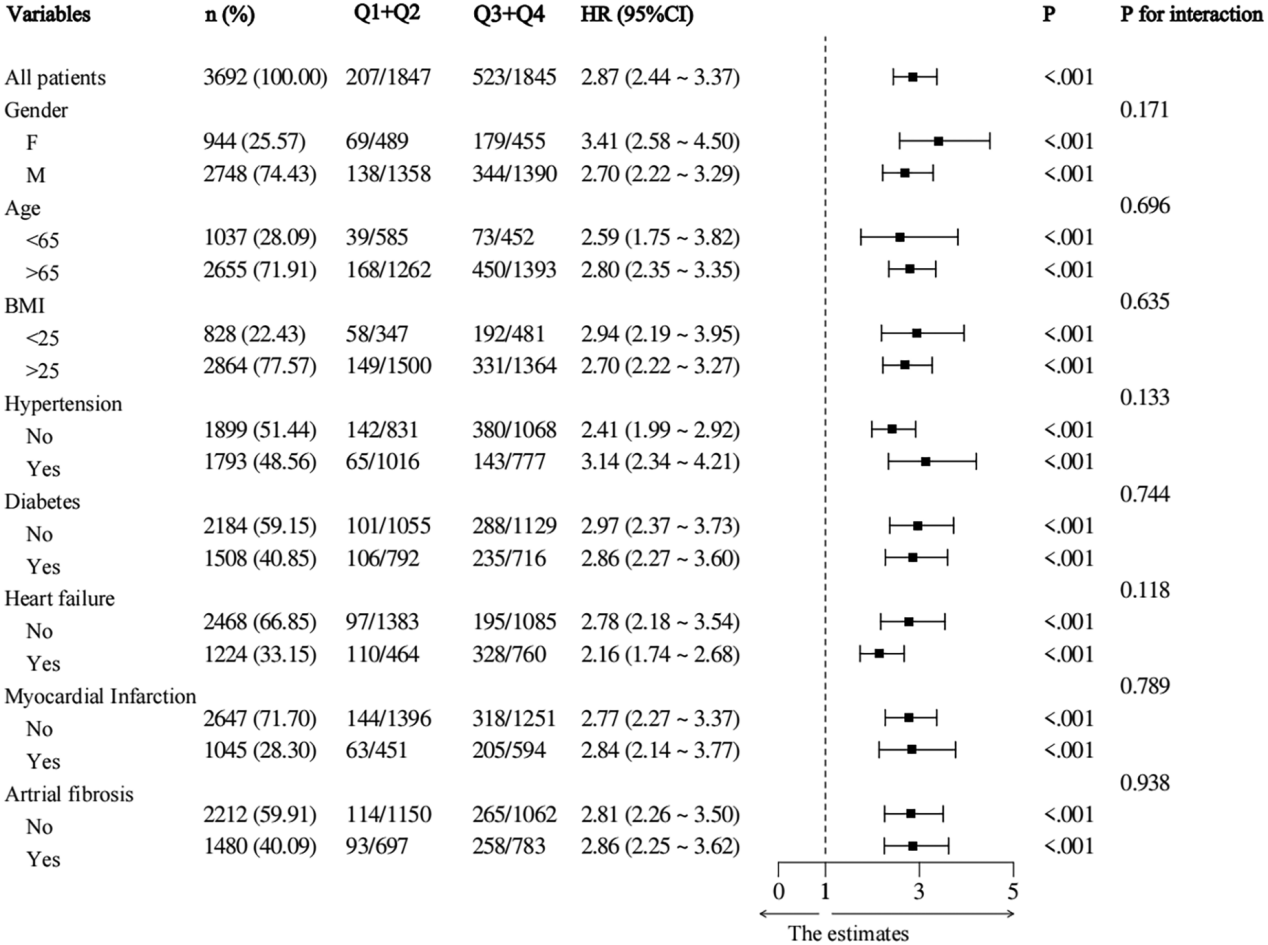
Subgroup analysis with 365-mortality as the outcome event in low and high NLR group (Q1 + Q2 vs. Q3 + Q4).

Furthermore, we conducted subgroup analyses of patient outcome events based on various risk factors including age, gender, BMI, AMI, AHF, hypertension, and diabetes. Based on the subgroup analysis examining 30-day mortality as the primary outcome event, a high NLR (Q3 + Q4 NLR > 5.54) in the subgroups demonstrated a significant association with primary 30-day mortality in patients diagnosed with critical CAD (Figure 4). Furthermore, the correlation between NLR and 365-day mortality was constant across all subgroups in our analysis, regardless of age (<65 and >65 years), sex, or BMI (Figure 5). Higher NLR levels are related to an increased risk of death, and the results were consistent in different subgroups according to comorbidities like hypertension, heart failure, and diabetes.

### Sensitivity analysis

**Figure 6:**
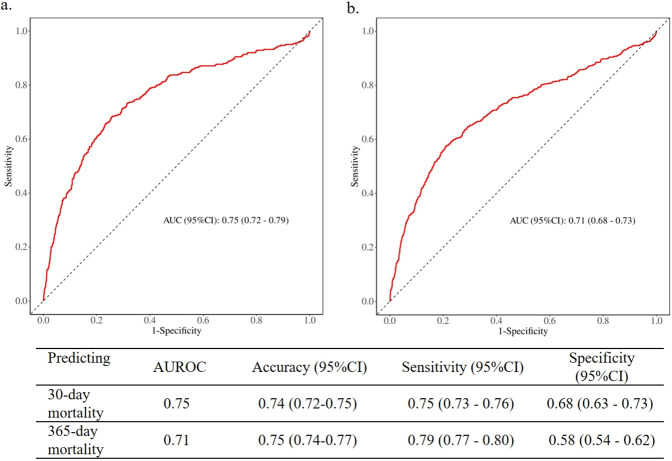
Receiver operating characteristic curves of NLR predicting 30-day and 365-day mortality. **(a)** 30-day mortality; **(b)** 365-day mortality.

The Receiver Operating Characteristic (ROC) analysis was employed to assess the discriminative capacity of NLR for detecting CAD in individuals. The findings indicated that the Area Under the ROC Curve (AUROC) for the NLR predicting 30-day mortality, when combined with various demographic and clinical variables, was 0.75 (95% CI: 0.72–0.79), with a sensitivity of 0.75 and a specificity of 0.68 (Figure 6b). Meanwhile, the AUROC for NLR predicting 365-day mortality, also combined with demographic and clinical variables, was 0.71 (95% CI: 0.68–0.73), with a sensitivity of 0.79 (Figure 6b). The result suggested NLR performed better in predicting short-term mortality than long-term mortality.

## Discussion

In this retrospective cohort study, we have demonstrated the presence of a statistically significant positive association between baseline NLR and short-term and long-term mortality among patients diagnosed with coronary artery disease. High NLR at ICU admission was associated with higher 30-day and 365-day mortality. We found a “U” relationship between NLR and the hazard ratio of outcome events in the RCS-based analyses, which is consistent with the results of the analyses described above. The stability of the relationship persisted following adjustments for demographic and clinical confounders, indicating a potential association between an early rise in NLR and unfavorable short-term and long-term outcomes in patients with CAD.

Inflammation plays a crucial role in the development and progression of CAD (13, 14). Modern research not only enhances interest in traditional inflammatory biomarkers such as hsCRP, but also raises awareness of a readily accessible and widely available simple biomarker within the clinical community (15, 16). One such biomarker is NLR, which is derived from the complete blood count. The NLR incorporates data from both the innate immune system, primarily mediated by neutrophils, and the adaptive immune response, facilitated by lymphocytes. Previous studies have shown that a high NLR is linked to atrial fibrillation, heart failure, and cardiac death in patients with cardiovascular diseases (17–19). Furthermore, studies have demonstrated the stability of NLR over time and its responsiveness to anti-inflammatory treatments, suggesting its potential utility as an alternative or adjunct inflammatory biomarker in clinical settings. Previous research has demonstrated that elevated NLR is correlated with a poor short-term and long-term prognosis in several conditions, such as tumors, sepsis, and ARDS (20). In the study, NLR levels were independently associated with an increased risk of all-cause mortality in the population. A recent meta-analysis comprising 23 studies involving patients with ACS revealed that an elevated NLR upon admission was significantly correlated with increased mortality rates and a higher incidence of major adverse clinical outcomes (21). In our study, relatively more abundant data was analyzed and indicated that the risk of cardiovascular events was not significant between quartiles 1 and 2 groups. This study conclusively illustrated that individual with high NLR (NLR > 5.54) exhibit increased cardiovascular risk and all-cause mortality compared to those with low NLR (NLR < 5.54), which varies from the knots implied in other studies. Timely risk stratification is crucial in the treatment of patients with coronary artery disease, and adherence to strict guideline-recommended therapies should be considered for this subset of higher-risk patients. While there is limited research on interventions for patients with high NLR and CAD, such treatments have the potential to mitigate their adverse cardiovascular outcomes.

The link between high NLR and cardiovascular events can be explained by various mechanisms. Both high neutrophil count and low lymphocyte count can impact the clinical outcomes of CAD patients. Neutrophils release substantial quantities of inflammatory mediators and modulate the inflammatory response (22). Moreover, neutrophils have been shown to increase the vulnerability of atherosclerotic plaques through the release of protective enzymes such as myeloperoxidase and superoxide radicals (3, 23). On the other hand, lymphocytes serve as a key regulatory component of the immune system, with studies indicating that inflammatory activation can induce apoptosis in these cells (24, 25). Additionally, a reduction in lymphocyte count has been identified as an early indicator of physiological stress and multivisceral failure resulting from myocardial ischemia, which is facilitated by the release of cortisol (26). Other studies have also found that certain components of white blood cells or platelets can indicate inflammation (27, 28). NLR levels may be a more accurate predictor of cardiovascular events compared to neutrophil levels alone, as neutrophils are linked to inflammation and cardiovascular diseases.

In conclusion, the findings of this study demonstrate a significant association between NLR and all-cause mortality in patients diagnosed with critical CAD. It is recommended that it is crucial to pay closer attention to neutrophil and lymphocytes fluctuations in patients during their ICU stay. Furthermore, due to the presence of numerous confounding variables in ICU patients, a comprehensive prospective study is warranted to further elucidate the relationship between NLR and adverse outcomes in individuals with critical CAD.

## Limitations

In this research, pertinent clinical data regarding patients diagnosed with CAD was obtained from the MIMIC-IV database. It should be noted that the complete clinical diagnostic information of the patients may not have been extracted, and there are potential confounding variables that could impact the overall mortality rates. Additionally, the potential relationship between NLR and adverse outcomes other than all-cause mortality was not explored in this study. Furthermore, the patient's lipid level was identified as an independent risk factor for an unfavorable prognosis; nevertheless, due to a substantial quantity of absent lipid data, these levels were not incorporated into the present study. Ejection fraction (EF) is indeed a crucial prognostic parameter in cardiovascular research. In our study, EF was not included in the baseline characteristics due to the missing of a significant proportion of patients. Moreover, the COX regression Model for NLR was computed utilizing the specific study cohort and may not be universally applicable to other populations. It is our contention that regression models should be developed using data sourced from diverse extensive databases to determine the NLR for distinct population subsets.

## Data Availability

The original contributions presented in the study are included in the article/Supplementary Material, further inquiries can be directed to the corresponding author.
