# Supplementary material for: Association between neutrophil to lymphocyte ratio and all-cause mortality in critical patients with coronary artery disease - a study based on the MIMIC-IV database
Source: Front Cardiovasc Med. 2025 Mar 21;12:1502964. doi: 10.3389/fcvm.2025.1502964 (PMC11968710; doi:10.3389/fcvm.2025.1502964)
Supplement: Supplementary file 1 [file Datasheet1.pdf]

Association between neutrophil to lymphocyte ratio and all-cause mortality in critical patients with coronary artery disease -A study based on the MIMIC-IV database

Yao Li [1†], Dongbo Chen [2†], Yifei Fan[1], Qing Zhu[1], Han Deng[3], Xin Chai[1#]

Table S1 Baseline characteristics of the Survivors and Non-survivors groups

Variables	Total (n = 3692)	Survivor (n = 2962)	Non-survivor (n = 730)	*P*
Age	70.64 ± 10.95	69.30 ± 10.47	76.08 ± 11.18	**<.001**
AGE (%)				**<.001**
<65	1037 (28.09)	925 (31.23)	112 (15.34)	
>65	2655 (71.91)	2037 (68.77)	618 (84.66)	
Gender (%)				**<.001**
Female	944 (25.57)	696 (23.50)	248 (33.97)	
Male	2748 (74.43)	2266 (76.50)	482 (66.03)	
BMI	29.54 ± 6.51	29.86 ± 6.14	28.25 ± 7.73	**<.001**
Group (%)				**<.001**
Q1	923 (25.00)	809 (27.31)	114 (15.62)	
Q2	923 (25.00)	831 (28.06)	92 (12.60)	
Q3	923 (25.00)	776 (26.20)	147 (20.14)	
Q4	923 (25.00)	546 (18.43)	377 (51.64)	
WBC (×10^9/L)	13.86 ± 8.39	13.83 ± 8.06	13.99 ± 9.64	0.668
RBC (×10^9/L)	3.46 ± 0.62	3.48 ± 0.59	3.38 ± 0.74	**<.001**
Neutrophil	10.29 ± 5.49	10.06 ± 4.87	11.20 ± 7.45	**<.001**
Lymphocytes	1.97 ± 4.74	2.12 ± 5.22	1.33 ± 1.64	**<.001**
Platelet	169.37 ± 72.15	165.64 ± 64.81	184.53 ± 94.92	**<.001**
Hemoglobin	10.34 ± 1.77	10.44 ± 1.70	9.93 ± 1.99	**<.001**
RDW (%)	14.28 ± 2.08	13.88 ± 1.72	15.89 ± 2.57	**<.001**
Hematocrit (%)	31.57 ± 5.15	31.72 ± 4.86	30.94 ± 6.14	**0.001**
Glucose	136.59 ± 51.30	130.21 ± 40.65	162.46 ± 76.03	**<.001**
SOFA	5.44 ± 3.12	5.07 ± 2.78	6.94 ± 3.89	**<.001**
APSIII	41.09 ± 19.51	37.51 ± 17.05	55.58 ± 22.01	**<.001**
SAPSII	38.66 ± 12.40	36.79 ± 11.24	46.24 ± 13.91	**<.001**
OASIS	31.58 ± 7.89	30.70 ± 7.39	35.17 ± 8.80	**<.001**
Charlson	5.37 ± 2.72	4.79 ± 2.38	7.74 ± 2.73	**<.001**
Hypertension (%)				**<.001**
No	1899 (51.44)	1377 (46.49)	522 (71.51)	
Yes	1793 (48.56)	1585 (53.51)	208 (28.49)	
DM (%)				**<.001**
No	2184 (59.15)	1795 (60.60)	389 (53.29)	
Yes	1508 (40.85)	1167 (39.40)	341 (46.71)	
AF (%)				**<.001**
No	2468 (66.85)	2176 (73.46)	292 (40.00)	
Yes	1224 (33.15)	786 (26.54)	438 (60.00)	
AMI (%)				**<.001**

Variables	Total (n = 3692)	Survivor (n = 2962)	Non-survivor (n = 730)	*P*
No	2647 (71.70)	2185 (73.77)	462 (63.29)	
Yes	1045 (28.30)	777 (26.23)	268 (36.71)	
AF (%)				**<.001**
No	2212 (59.91)	1833 (61.88)	379 (51.92)	
Yes	1480 (40.09)	1129 (38.12)	351 (48.08)	
Invasive ventilation (%)				**<.001**
No	3654 (98.97)	2941 (99.29)	713 (97.67)	
Yes	38 (1.03)	21 (0.71)	17 (2.33)	
CABG (%)				**<.001**
No	2664 (72.16)	1997 (67.42)	667 (91.37)	
Yes	1028 (27.84)	965 (32.58)	63 (8.63)	
PTCA (%)				0.178
No	3679 (99.65)	2954 (99.73)	725 (99.32)	
Yes	13 (0.35)	8 (0.27)	5 (0.68)	
Hospital day	9.49 ± 8.37	8.83 ± 7.11	12.13 ± 11.85	<.001
ICU day	3.08 ± 4.25	2.66 ± 3.35	4.77 ± 6.52	<.001

Data are expressed in n (%) and median (inter-quartile range).

CABG: coronary artery bypass grafting, PTCA: Percutaneous transluminal coronary angioplasty, AMI: acute myocardial infarction, AHF: acute heart failure, AF: atrial fibrillation, SOFA: Sepsis-Organ Failure Assessment Score, APSIII: Acute Physiology Score III, SAPSII: Simplified Acute Physiology Score, OASIS: Oxford Acute Severity of Illness Score, WBC: white blood cells, RBC: red blood cells, PLT: platelets, HGB: hemoglobin, FPG: fasting plasma glucose, Hct: Hematocrit

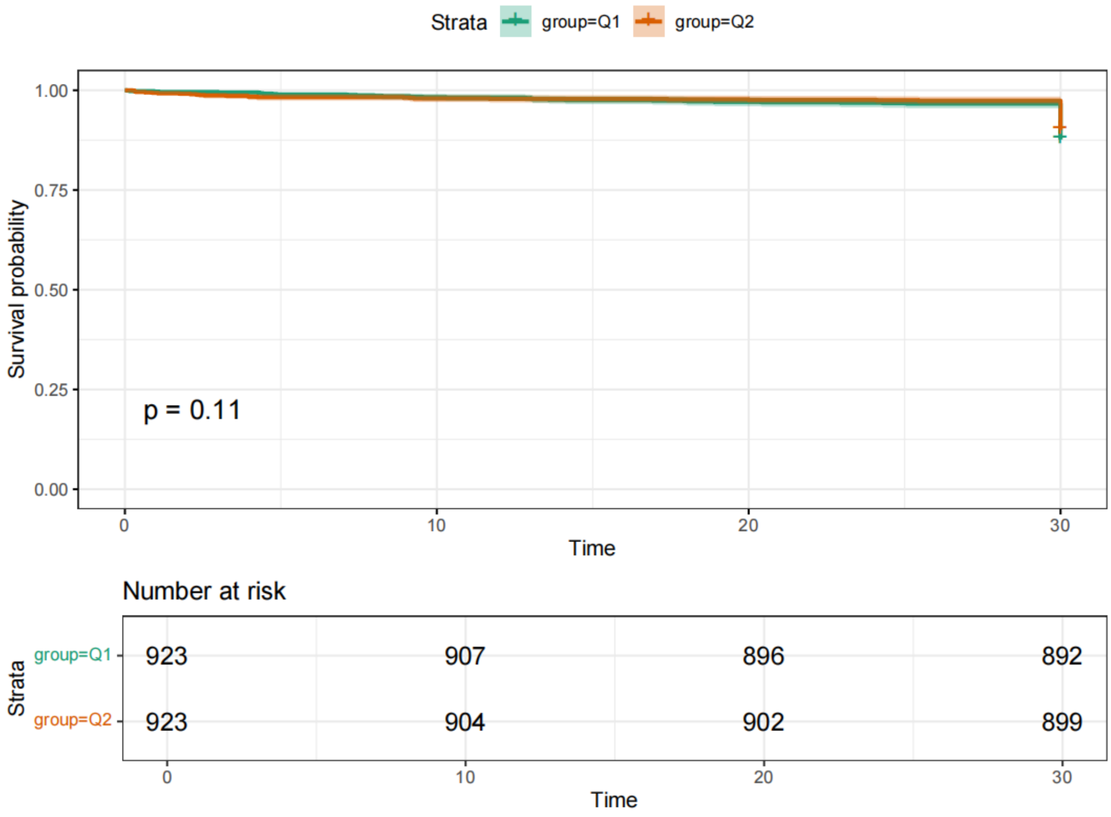

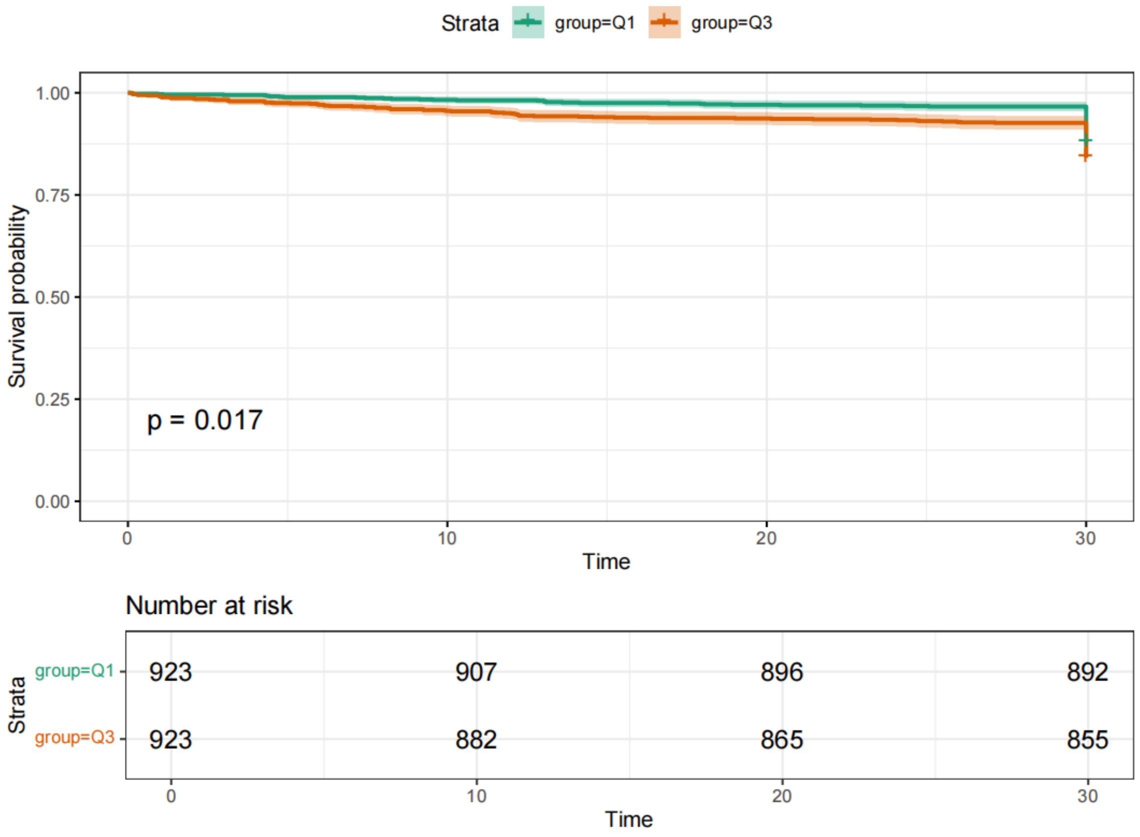

Strata group=Q1 group=Q3
Survival probability
1.00
0.75
0.50
0.25
0.00
p = 0.017
0
10
20
30
Time
Number at risk
Strata
group=Q1 923 907 896 892
group=Q3 923 882 865 855
0
10
20
30
Time

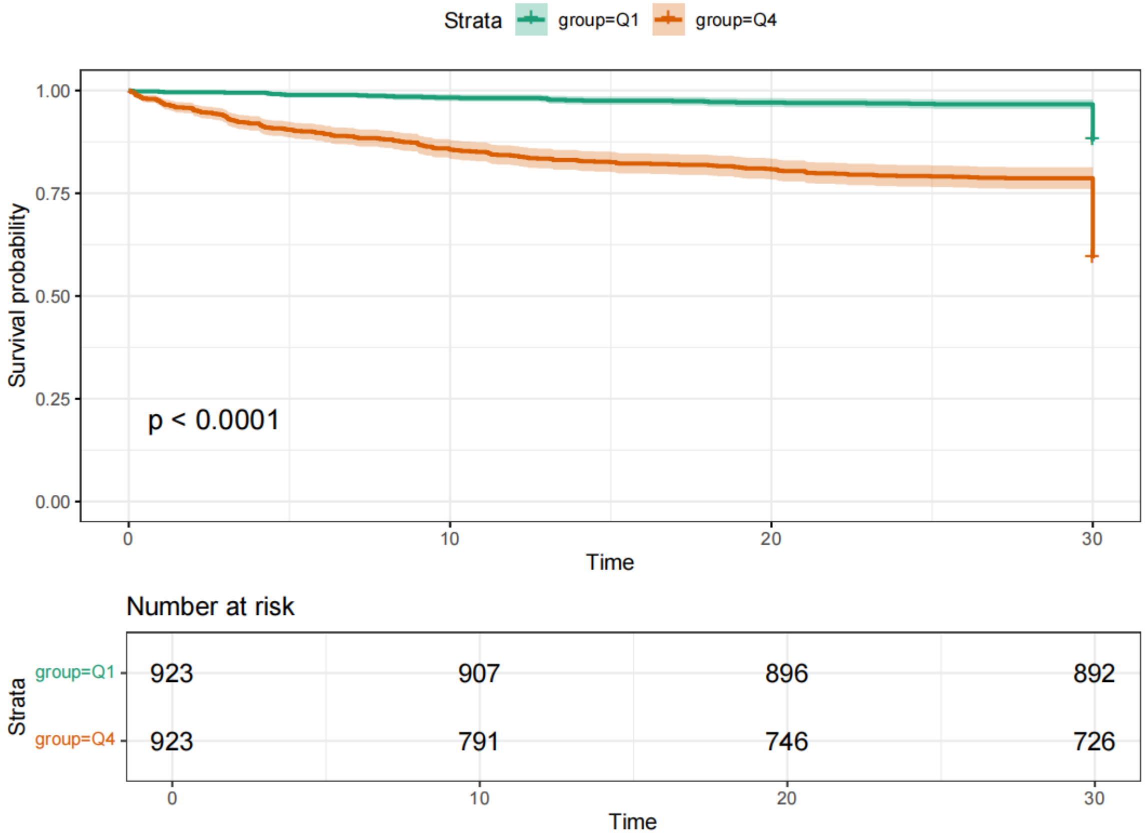

Strata group=Q1 group=Q4
Survival probability
1.00
0.75
0.50
0.25
0.00
p < 0.0001
0
10
20
30
Time
Number at risk
Strata
group=Q1 923 907 896 892
group=Q4 923 791 746 726
0
10
20
30
Time

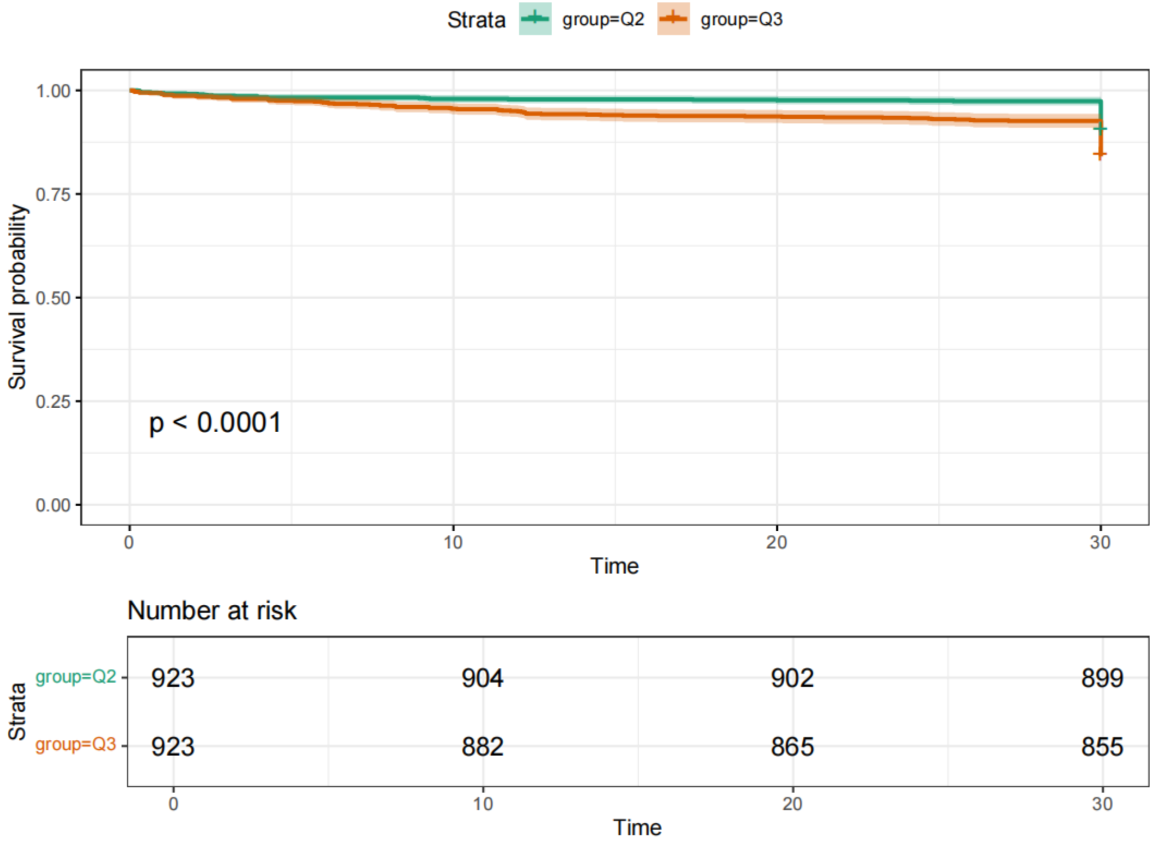

Figure S1 Kaplan-Meier all-cause mortality survival analysis curve : showing comparison of mortality within 30 days between every two groups respectively.

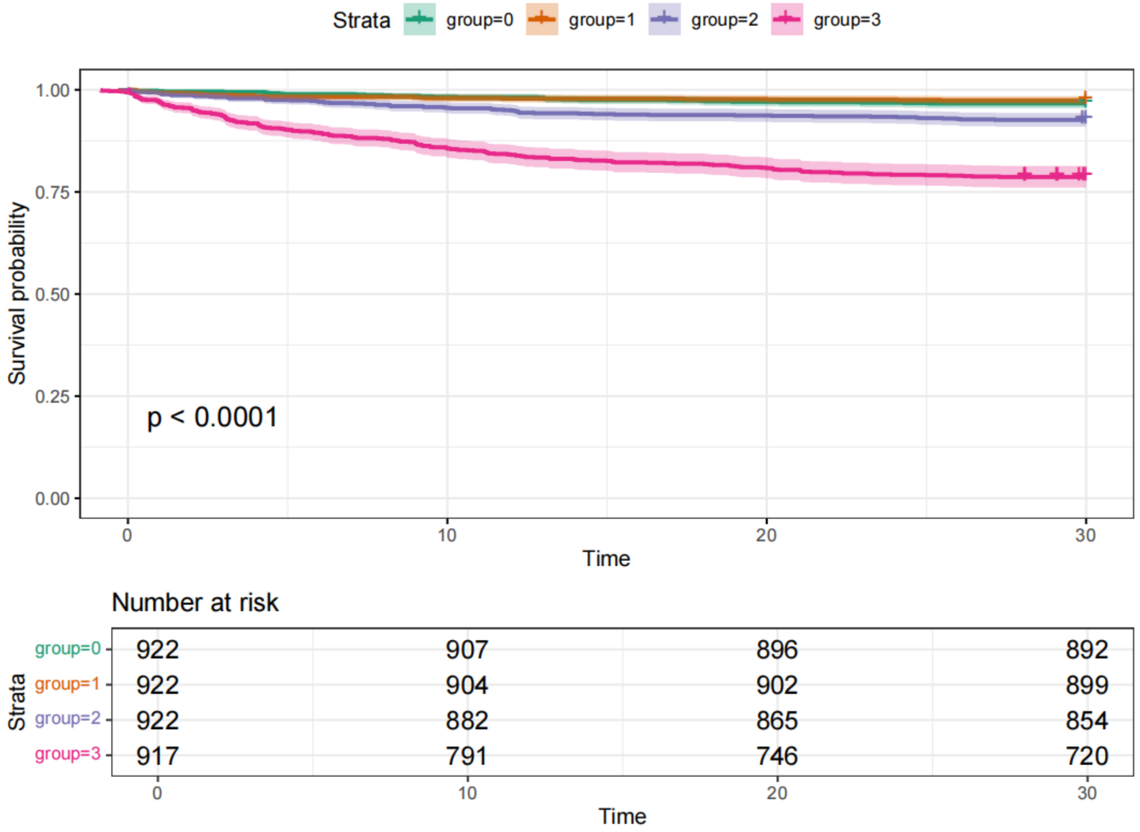

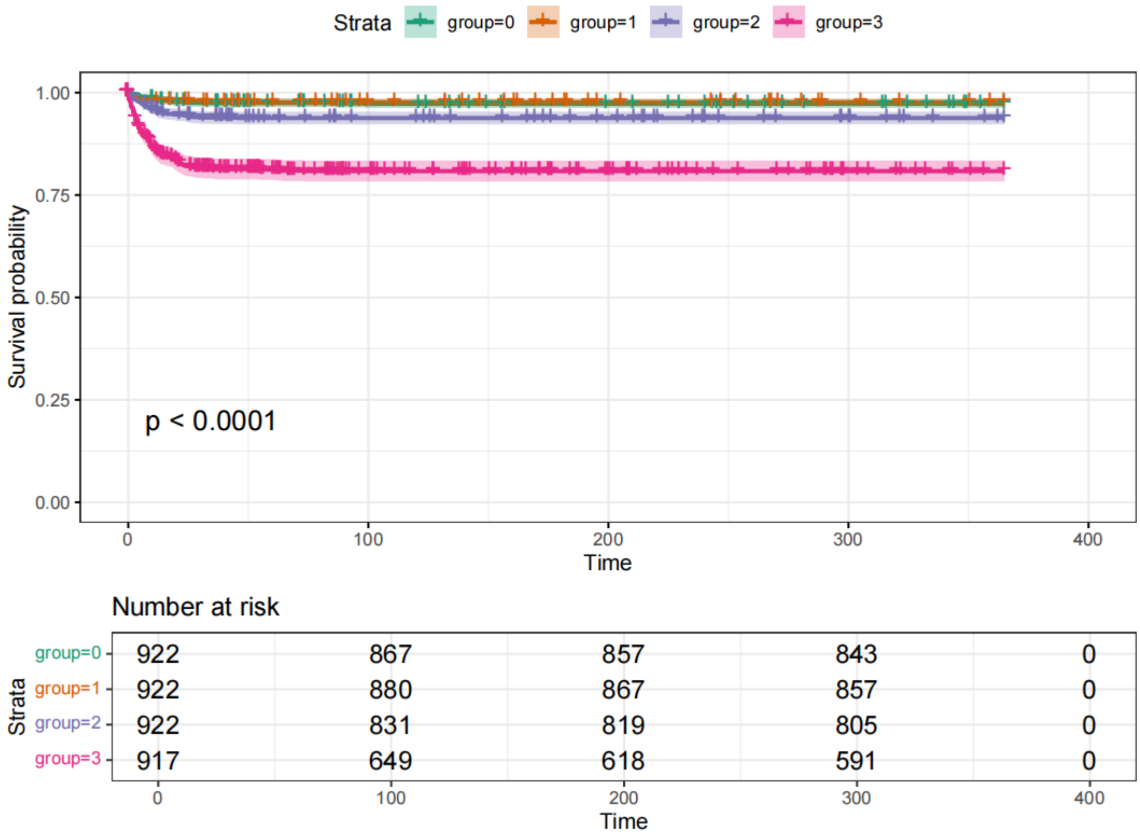

Figure S2 Kaplan-Meier all-cause mortality survival analysis curve: showing comparison of mortality within 30 days and 365 days between quantiles of creatinine (group 0 (n = 923, Scr ≤ 0.8), group1 (n =923, 0.8 < Scr ≤ 0.97), group2 (n = 923, 0.97 < Scr ≤ 1.3), and group3 (n = 923, Scr > 1.3)).

Table S2

Variables	All-cause mortality within 30 days		All-cause mortality within 365 days	
	HR (95%CI)	*P*	HR (95%CI)	*P*
Group 0	1.00 (Reference)		1.00 (Reference)	
Group 1	0.78 (0.46~1.32)	0.351	0.88 (0.50~1.56)	0.667
Group 2	2.24 (1.47~3.43)	<.001	2.33 (1.46~3.73)	<.001
Group 3	7.06 (4.84~10.31)	<.001	7.75 (5.10~11.79)	<.001

HR: Hazards Ratio, CI: Confidence Interval

Table S2 Multivariate Cox regression analysis between creatinine quantiles and short-term and long-term survival, with the group 0 serving as the reference category. Adjust: age, gender, BMI

Table S3

Subgroup analysis with 30-mortality as the outcome event

Variables	n (%)	G1	G2	HR (95%CI)	*P*	P for interaction
All patients	3692 (100.00)	56/1847	271/1845	5.15 (3.86 ~ 6.87)	**<.001**	
Cabg						0.025
0	2664 (72.16)	48/1185	265/1479	4.77 (3.50 ~ 6.48)	**<.001**	
1	1028 (27.84)	8/662	6/366	1.36 (0.47 ~ 3.91)	0.572	

Subgroup analysis with 365-mortality as the outcome event

Variables	n (%)	G1	G2	HR (95%CI)	*P*	P for interaction
All patients	3692 (100.00)	160/1847	467/1845	3.27 (2.73 ~ 3.91)	**<.001**	
Cabg						<.001
0	2664 (72.16)	129/1185	451/1479	3.21 (2.64 ~ 3.91)	**<.001**	
1	1028 (27.84)	31/662	16/366	0.93 (0.51 ~ 1.71)	0.823	

HR: Hazard Ratio, CI: Confidence Interval

Table S3 Subgroup analysis with 30-mortality and 365-mortality as the outcome event in low and high NLR group (Q1+Q2 VS Q3+Q4)

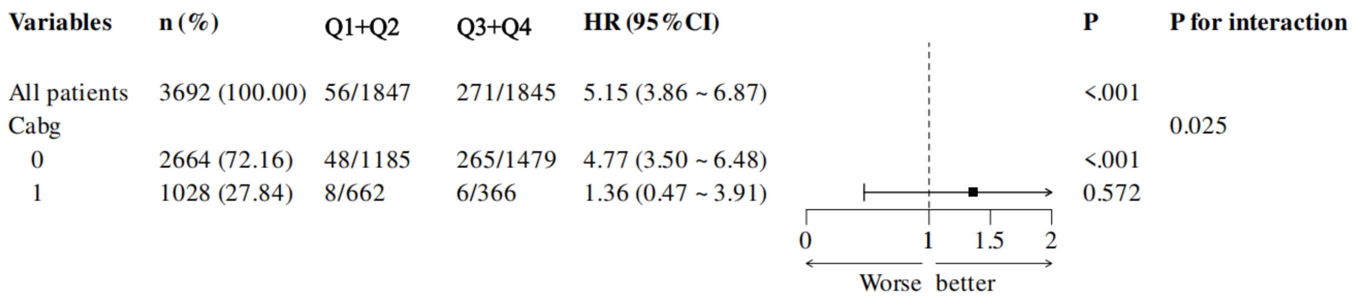

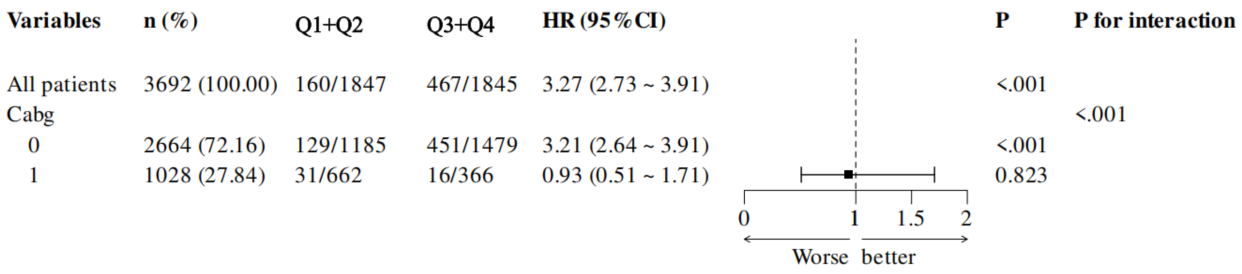

Figure S3 Forest subgroup analysis with 30-mortality and 365-mortality as the outcome event in low and high NLR group (Q1+Q2 VS Q3+Q4)
